# Impact of Coverage with an Acellular Dermal Matrix and Suturing Versus Primary Closure on Tongue Pain and Oral Morbidity After Lingual Mucosa Harvesting for Urethroplasty: A Retrospective Cohort Study

**DOI:** 10.1016/j.euros.2025.11.010

**Published:** 2025-12-10

**Authors:** Changhao Hou, Zhenwei Yu, Ziwen Liu, Tao Liang, Xuxiao Ye, Jiemin Si, Weidong Zhu, Chongrui Jin, Zhenghao Dai, Yinglong Sa, Lujie Song, Qiang Fu

**Affiliations:** a Department of Urology, Shanghai Sixth People's Hospital Affiliated to Shanghai Jiao Tong University School of Medicine, Shanghai 200233, China; b Shanghai Eastern Institute of Urologic Reconstruction, Shanghai 200233, China; c Shanghai Jiao Tong University School of Medicine, Shanghai 200233, China

**Keywords:** Urethral stricture, Lingual mucosal graft, Urethroplasty, Acellular dermal matrix, Pain, Oral morbidity

## Abstract

**Background and objective:**

Surgical management of the harvest site for lingual mucosal graft (LMG) urethroplasty remains controversial. Our aim was to compare pain intensity and oral morbidity at the donor site between primary closure (PC) and use of an artificial acellular dermal matrix (ADM) for coverage during suturing.

**Methods:**

We conducted a retrospective study of patients who underwent LMG urethroplasty between October 15, 2015, and December 18, 2022. Follow-up was conducted prospectively in the early postoperative period (7 d after surgery) and retrospectively for the 3-mo time point. Patients completed a postoperative questionnaire regarding LMG harvesting, including questions on pain intensity and oral morbidity. Propensity score matching was used to balance the PC and ADM groups, and generalized linear mixed models were used to evaluate factors influencing lingual pain (primary endpoint) and oral morbidity (secondary endpoint).

**Key findings and limitations:**

A total of 250/448 patients (55.8%) completed the postoperative tongue health questionnaire, including 174 in the PC group and 76 in the ADM group. ADM use for coverage during suturing significantly reduced early-stage pain (60.94% vs 92.80%; *p* < 0.001) and swelling (64.06% vs 88.80%; *p* < 0.001). The 3-mo data showed no significant between-group differences in oral morbidity, including pain, bleeding, swelling, numbness, salivary secretion, taste changes, and dietary and speech impairments. The retrospective design is the main study limitation.

**Conclusions and clinical implications:**

ADM use for coverage during suturing reduces early pain and swelling after LMG harvesting for urethroplasty in comparison to PC, with comparable long-term oral morbidity. Patients may benefit from ADM use in terms of faster early recovery.

**Patient summary:**

We compared two methods for suturing the tongue after taking tissue to repair a defect in the urethra. Our results show that use of product called an artificial acellular dermal matrix (ADM) significantly reduces early pain and swelling after surgery in comparison to the traditional method. Long-term results are comparable for the two methods. Patients may benefit from ADM use in terms of faster early recovery.

## Take Home Message

In comparison to primary closure of the donor site, coverage with an acellular dermal matrix and suturing reduces early pain and swelling after urethroplasty with a lingual mucosa graft, with comparable 3-mo oral morbidity. Artificial acellular dermal matrix use may improve early-stage recovery in this setting.

## 1 Introduction

Urinary tract repair is required in various clinical scenarios, the most common of which are urethral stricture and hypospadias [1]. For long-segment urethral defects, end-to-end urethral anastomosis is often not feasible, and treatment may require the use of a skin flap or free graft. Advances in repair techniques mean that oral mucosa grafting has become one of the preferred options for urethroplasty, with a lingual mucosa graft (LMG) and buccal mucosa graft (BMG) the most commonly used [2]. BMGs and LMGs share similar tissue characteristics, but LMGs offer advantages such as ease of harvest, resistance to infection, favorable tissue properties (thick epithelium, high elastic fiber content, thin lamina propria, and rich vascularization), and excellent adaptability to moist environments [3], [4]. Particularly for patients with long-segment urethral strictures, the tongue can serve as a reservoir of tissue for extensive defects [5].

However, LMG use for urethral repair, which involves sacrificing the donor site, is not without limitations. Numerous studies have focused on the oral morbidity associated with LMG harvesting, such as numbness, pain, and motor dysfunction [6], [7]. Surgical management of the LMG harvest site in patients undergoing LMG urethroplasty (LMGU) still lacks consensus [8], [9]. Whether to suture the lingual mucosa defect area is a subject of ongoing debate [1], [7]. The advantages of primary suturing include hemostasis and promotion of wound healing, but this practice may lead to compression of the tongue muscles. Suturing of the donor site could potentially result in future difficulties in terms of tongue movement dysfunction, taste impairment, and speech disorders [7], [10]. Conversely, although some studies have shown that keeping the donor site open can reduce postoperative pain, pain incidence remains high, and it has not been reported whether additional repair of the tongue defect is necessary in such cases [11], [12].

While there are numerous articles on donor-site morbidity following LMGU, none has described the impact of donor-site repair on oral pain and morbidity. Successful urethral stricture repair via LMGU while minimizing donor-site complications and alleviating patients’ psychological fears is a topic worthy of exploration. With advances in tissue engineering, biomaterials provide a new alternative in this setting. Simonato et al [13] demonstrated that use of an artificial acellular dermal matrix (ADM) for BMG repair was a safe and feasible approach. However, in comparison to primary closure (PC), ADM use showed no significant advantages for either early or long-term oral morbidity [14]. Furthermore, it remains unclear whether ADM use is beneficial for LMG repair. Therefore, we compared the effects of PC of the donor site versus ADM use for coverage during suturing on postoperative pain and morbidity at the LMG harvest site for patients undergoing LMGU.

## 2 Patients and methods

This retrospective study was approved by the ethics committee of Shanghai Sixth People’s Hospital (reference 2022-KY-065). The surgical technique used for LMGU was in accordance with local resource availability in two distinct periods. During 2015–2018, PC repair was used for all patients undergoing LMGU, as ADMs were not available in the hospital at that time. Following the introduction of ADM material (Lando, Shenzhen Lando Biomaterials, Shenzhen, China; 5 cm × 5 cm; derived from bovine Achilles tendon, composed primarily of type I collagen and chondroitin sulfate) in the hospital, LMGU was performed with ADM repair (ADM group, 2019–2022). All surgeries were performed by four senior urologists, all of whom have more than 10 yr of urethroplasty experience and perform more than 30 LMGU procedures per year. The inclusion criteria were male patients older than 18 yr who underwent LMGU at our hospital between October 15, 2015, and December 18, 2022. Exclusion criteria were individuals with known or suspected concomitant oral diseases (eg, gingivitis and dental caries), psychiatric disorders, cognitive impairments, or chronic pain conditions.

### 2.1 Surgical technique

**Fig. 1:**
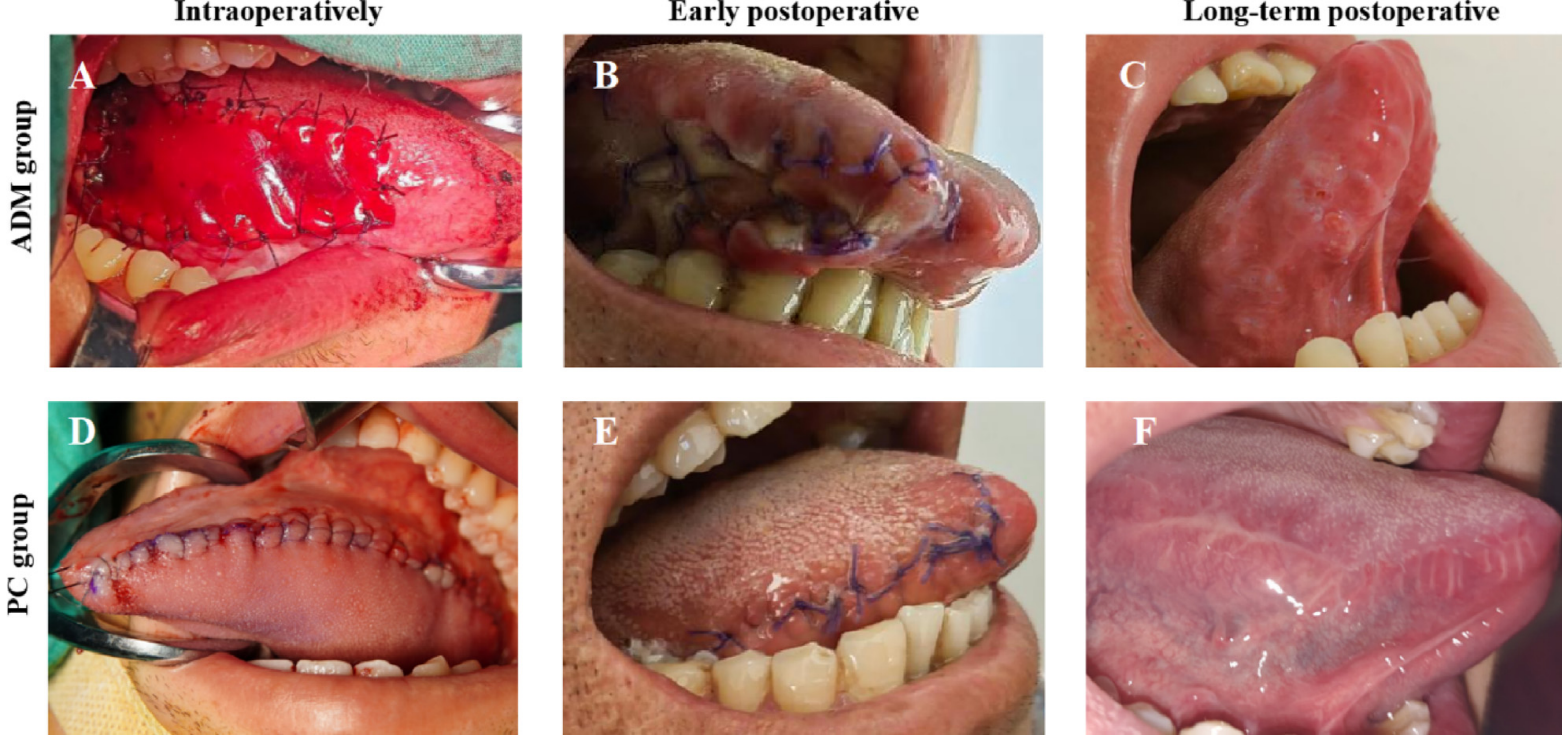
Images of lingual defects in the ADM and PC groups during surgery, in the early postoperative period (7 d after surgery), and at 3-mo follow-up. (A) Intraoperative LMG repair with ADM. Lingual appearance in the ADM group at (B) 7 d and (C) 3 mo. (D) Primary closure of the lingual mucosal defect. Lingual appearance in the PC group at (E) 7 d and (F) 3 mo. ADM = acellular dermal matrix; PC = primary closure.

For each patient, an oral examination was conducted 1 wk before surgery to exclude oral mucosal diseases. Oral preparation involved the use of mouthwash for 3 d. After accurate measurement of the length of the urethral defect, intraoral disinfection was performed. A mouth retractor was used to expose the oral cavity, and the tongue was pulled out of the mouth and the tip was sutured. The LMG for harvesting was marked on the ventral side of the tongue. A solution of 1:100 000 epinephrine in saline was injected submucosally to expand the submucosal layer, and the required length of mucosa was excised. If a longer graft (>8–10 cm) was needed, bilateral graft harvesting was performed. The donor site was closed with 4-0 absorbable sutures. In the PC group, the donor area was closed using continuous sutures. In the ADM group, the defect was covered with an appropriately sized ADM patch that was secured to the edges of the tongue defect with interrupted sutures, with reinforcement of the central area of the defect via additional sutures (Fig. 1).

### 2.2 Postoperative management

On day 2 after surgery, patients were allowed to resume a cold liquid diet, gradually transitioning to a semi-liquid and then a regular diet. For the first week postoperatively, patients were advised to avoid hard and irritating foods. After each meal, patients were instructed to rinse their mouths with chlorhexidine solution (Corsodyl) or saline for 3–5 d. Once the absence of bleeding at the LMG donor site was confirmed, patients began tongue function training, which included exercises for tongue movement, pronunciation, and chewing.

### 2.3 Patient questionnaire

Patients were asked to complete a two-part post-LMGU tongue health questionnaire. The first part covers the early stage (7 d) after surgery and the second part assesses outcomes at 3 mo. As tongue function is not fully restored in the early postoperative period, the early-stage questionnaire primarily focuses on sensory indicators such as pain, swelling, and bleeding. The 3-mo questionnaire also incorporates functional assessments, including mouth opening, taste, salivary secretion, oral sensation, diet, and speech. The nature of oral pain was evaluated using the Short-Form McGill Pain Questionnaire-Present Pain Intensity [15]. Questions regarding oral morbidity were scored using a 5-point scale: 0 = None at all; 1 = Slight; 2 = Moderate; 3 = Severe; and 4 = Very severe. Data for patient-reported outcomes were collected via two sources. Data for postoperative day 7 were prospectively collected during the perioperative period. The 3-mo postoperative data were collected retrospectively during the study enrollment phase by asking patients to recall their symptoms and oral function.

### 2.4 Statistical analysis

To balance baseline characteristics between the two groups, propensity score matching was applied to the follow-up cases at a 1:2 PC/ADM ratio to select a cohort for analysis. The primary endpoint was the intensity of tongue pain at the LMG harvest site. Secondary endpoints included the incidence of oral morbidity related to the tongue in the two groups. Results for continuous variables with a normal distribution are reported as the mean ± standard deviation and were compared between the groups using independent Student’s *t* tests. Results for categorical variables are reported as the frequency and proportion, and were compared between the groups using a Pearson χ^2^ test or Fisher’s exact test, as appropriate. A generalized linear mixed model (GLMM) approach was used to analyze dependent variables. All tests were two-sided, and *p* < 0.05 was considered statistically significant. All analyses were performed using SPSS version 26 (IBM Corp., Armonk, NY, USA).

## 3 Results

### 3.1 Baseline characteristics and clinical features of the study population

**Fig. 2:**
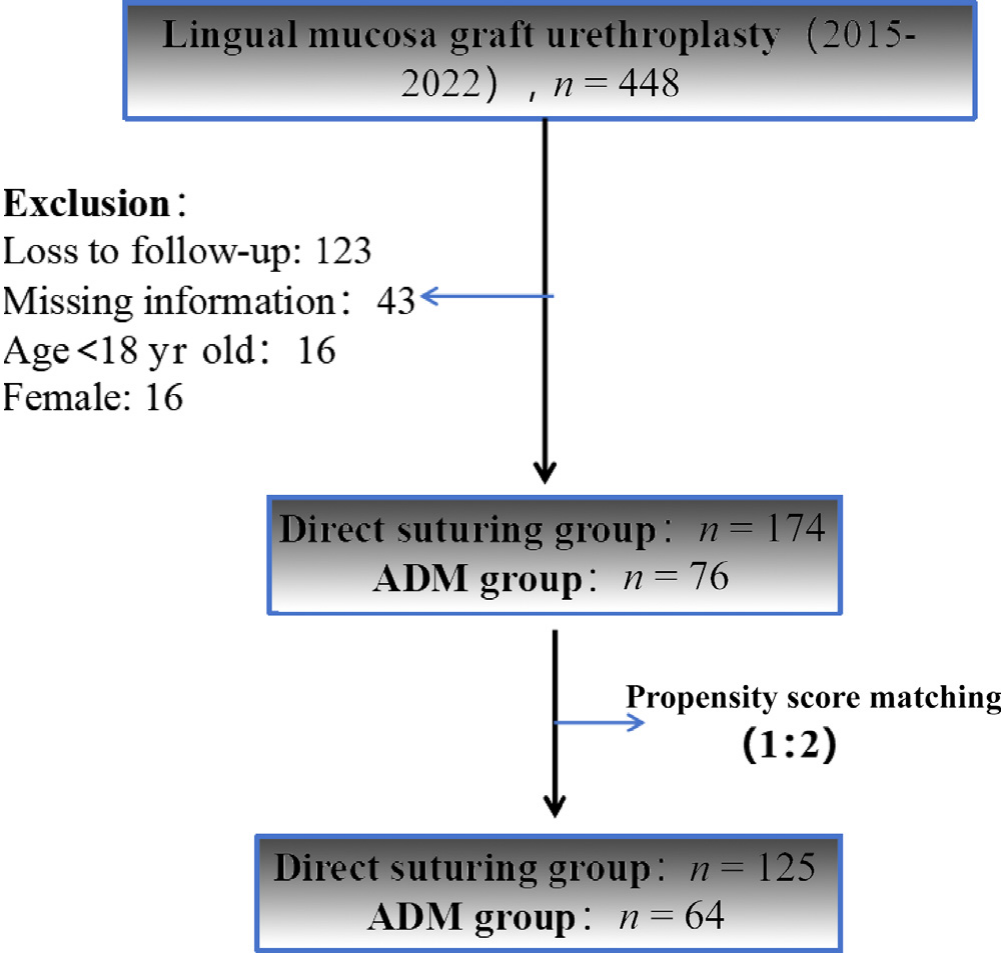
Study flowchart. ADM = acellular dermal matrix.

Retrospective postoperative follow-up was conducted for 448 patients with contact information available who underwent LMGU at our hospital between 2015 and 2022. A total of 250 patients completed the post-LMGU tongue health questionnaire, of whom 174 were in the PC group and 76 were in the ADM group (Fig. 2). After propensity score matching, 189 patients were included in the analysis, of whom 125 were in the PC group and 64 were in the ADM group (Fig. 2).

The mean patient age was 43.88 ± 15.64 yr and the mean stricture length was 5.17 ± 2.63 cm. The most common stricture etiologies were iatrogenic, traumatic injury, and hypospadias. The mean LMG length harvested was 5.35 ± 2.74 cm (Table 1). Most strictures were located in the penile urethra, and more than half of the patients had no prior surgical history. There were no significant differences between the groups in age, stricture length, harvest site location, LMG length, urethral stricture length, location [16], and etiology, or perioperative analgesic use (all *p* > 0.05).

**Table 1:** Patient characteristics at baseline

Parameter^a^	Overall cohort (*n* = 189)	PC group (*n* = 125)	ADM group (*n* = 64)	*p* value
Age (yr)	43.88 ± 15.64	43.61 ± 15.24	44.39 ± 16.50	0.748^c^
Time to diagnosis (mo)	11.40 ± 12.00	11.37 ± 10.94	11.45 ± 13.94	0.965^c^
Urethral stricture length (cm)	5.17 ± 2.63	5.28 ± 2.74	4.95 ± 2.42	0.415^c^
LMG length (cm)	5.35 ± 2.74	5.50 ± 2.88	5.06 ± 2.44	0.296^c^
LMG width (cm)	1.44 ± 0.31	1.45 ± 0.33	1.42 ± 0.28	0.541^c^
Follow-up (d)	1072.80 ± 3279.20	988.55 ± 831.91	1237.34 ± 5539.41	0.623^c^
Etiology, *n* (%)				0.881^d^
Iatrogenic	49 (25.93)	35 (28.00)	14 (21.88)	
Congenital	4 (2.12)	3 (2.40)	1 (1.56)	
Traumatic	42 (22.22)	25 (20.00)	17 (26.56)	
Lichen sclerosus	20 (10.58)	14 (11.20)	6 (9.38)	
Idiopathic/unknown	27 (14.29)	16 (12.80)	11 (17.19)	
Failed hypospadias repair	29 (15.34)	20 (16.00)	9 (14.06)	
Hypospadias	18 (9.52)	12 (9.60)	6 (9.38)	
Inflammatory	0	0	0	
Previous interventions, *n* (%)				0.888^d^
None	106 (56.08)	69 (55.20)	37 (57.81)	
Minimally invasive	33 (17.46)	23 (18.40)	10 (15.62)	
Urethroplasty	50 (26.46)	33 (26.40)	17 (26.56)	
Urethral stricture location, *n* (%)^b^				0.140^e^
S1	39 (20.63)	27 (21.60)	12 (18.75)	
S2a	9 (4.76)	9 (7.20)	0 (0.00)	
S2b	104 (55.03)	65 (52.00)	39 (60.94)	
S2c	6 (3.17)	4 (3.20)	2 (3.12)	
S2d	16 (8.47)	10 (8.00)	6 (9.38)	
S2d + S1	3 (1.59)	2 (1.60)	1 (1.56)	
S2d + S2b	4 (2.12)	1 (0.80)	3 (4.69)	
S3	8 (4.23)	7 (5.60)	1 (1.56)	
LMG harvest site, *n* (%)				0.617^d^
Left	52 (27.51)	37 (29.60)	15 (23.44)	
Right	95 (50.26)	60 (48.00)	35 (54.69)	
Bilateral	42 (22.22)	28 (22.40)	14 (21.88)	

ADM = acellular dermal matrix; LMG = lingual mucosa graft; PC = primary closure.

a Results for continuous variables are reported as the mean ± standard deviation.

b Trauma and Urologic Reconstruction Network of Surgeons classification system for anterior urethral stricture [16].

c Student’s *t* test.

d χ^2^ test.

e Fisher’s exact test.

### 3.2 Postoperative tongue pain intensity and morbidity in the ADM and PC groups

#### 3.2.1 Postoperative tongue pain intensity

In the early postoperative period, tongue pain was primarily mild in severity. There was a significant difference in tongue pain intensity between the groups: the ADM group reported lower incidence of pain than the PC group (*p* < 0.001). The 3-mo results showed that the incidence of pain had significantly decreased in both groups over time, with only 8.47% of patients reporting mild pain and no patients experiencing moderate or severe pain. Although the ADM group had a slightly lower incidence of pain than the PC group at 3 mo (7.81% vs 9.60%; *p* = 1.00), the difference was not statistically significant (Table 2).

**Table 2:** Oral morbidity results

Parameter	Patient response, *n* (%)			*p* value
	Overall cohort (*n* = 189)	PC group (*n* = 125)	ADM group (*n* = 64)	
**Early follow-up**				
Tongue bleeding				0.476^a^
None at all	78 (41.27)	49 (39.20)	29 (45.31)	
Slight	93 (49.21)	64 (51.20)	29 (45.31)	
Moderate	15 (7.94)	11 (8.80)	4 (6.25)	
Severe	3 (1.59)	1 (0.80)	2 (3.12)	
Very severe	0	0	0	
Tongue pain				<0.001^b^
No pain	34 (17.99)	9 (7.20)	25 (39.06)	
Mild	79 (41.80)	54 (43.20)	25 (39.06)	
Discomforting	58 (30.69)	48 (38.40)	10 (15.62)	
Distressing	17 (8.99)	14 (11.20)	3 (4.69)	
Horrible	1 (0.53)	0	1 (1.56)	
Excruciating	0	0	0	
Tongue swelling				<0.001^b^
None at all	37 (19.58)	14 (11.20)	23 (35.94)	
Slight	85 (44.97)	55 (44.00)	30 (46.88)	
Moderate	46 (24.34)	37 (29.60)	9 (14.06)	
Severe	18 (9.52)	16 (12.80)	2 (3.12)	
Very severe	3 (1.59)	3 (2.40)	0	
**Long-term follow-up**				
Tongue bleeding				1.000^a^
None at all	189 (100)	125 (100)	64 (100)	
Slight	0	0	0	
Moderate	0	0	0	
Severe	0	0	0	
Very severe	0	0	0	
Tongue pain				1.000^a^
No pain	172 (91.01)	113 (90.40)	59 (92.19)	
Mild	16 (8.47)	11 (8.80)	5 (7.81)	
Discomforting	1 (0.53)	1 (0.80)	0	
Distressing	0	0	0	
Horrible	0	0	0	
Excruciating	0	0	0	
Tongue swelling				0.845^a^
None at all	175 (92.59)	116 (92.80)	59 (92.19)	
Slight	13 (6.88)	8 (6.40)	5 (7.81)	
Moderate	1 (0.53)	1 (0.80)	0	
Severe	0	0	0	
Very severe	0	0	0	
Tongue numbness				0.401^a^
None at all	146 (77.25)	99 (79.20)	47 (73.44)	
Slight	39 (20.63)	23 (18.40)	16 (25.00)	
Moderate	2 (1.06)	2 (1.60)	0	
Severe	1 (0.53)	1 (0.80)	0	
Very severe	1 (0.53)	0	1 (1.56)	
Impairment of eating and drinking				0.603^a^
None at all	166 (87.83)	111 (88.80)	55 (85.94)	
Slight	19 (10.05)	12 (9.60)	7 (10.94)	
Moderate	3 (1.59)	1 (0.80)	2 (3.12)	
Severe	1 (0.53)	1 (0.80)	0	
Very severe				
Alteration of taste perception				0.271^a^
None at all	170 (89.95)	115 (92.00)	55 (85.94)	
Slight	18 (9.52)	9 (7.20)	9 (14.06)	
Moderate	1 (0.53)	1 (0.80)	0	
Severe	0	0	0	
Very severe	0	0	0	
Alteration of salivation				0.695^a^
None at all	174 (92.06)	115 (92.00)	59 (92.19)	
Slight	13 (6.88)	8 (6.40)	5 (7.81)	
Moderate	2 (1.06)	2 (1.60)	0	
Severe	0	0	0	
Very severe	0	0	0	
Speaking disorders				0.132^a^
None at all	146 (77.25)	100 (80.00)	46 (71.88)	
Slight	40 (21.16)	22 (17.60)	18 (28.12)	
Moderate	3 (1.59)	3 (2.40)	0	
Severe	0	0	0	
Very severe	0	0	0	
Difficulty in tongue protrusion				0.309^a^
None at all	169 (89.42)	114 (91.20)	55 (85.94)	
Slight	15 (7.94)	9 (7.20)	6 (9.38)	
Moderate	3 (1.59)	1 (0.80)	2 (3.12)	
Severe	1 (0.53)	1 (0.80)	0	
Very severe	1 (0.53)	0	1 (1.56)	
Tongue is skewed or retracted				0.158^a^
None at all	157 (83.07)	107 (85.60)	50 (78.12)	
Slight	28 (14.81)	17 (13.60)	11 (17.19)	
Moderate	4 (2.12)	1 (0.80)	3 (4.69)	
Severe	0	0	0 (0.00)	
Very severe	0	0	0	

ADM = acellular dermal matrix; PC = primary closure.

a Fisher’s exact test.

b χ^2^ test.

#### 3.2.2 Oral morbidity after LMGU

Table 2 shows the incidence of oral morbidity related to the tongue following LMGU. In the early postoperative period, there was a significant difference in tongue swelling between the two groups (*p* < 0.001), with mild swelling being the predominant issue. However, there was no significant difference in tongue bleeding between the groups at 7 d (*p* = 0.476). The 3-mo results indicate that numbness (20.80% vs 26.56%) and speech impairment (20.00% vs 28.12%) were the most common morbidity reported by patients in both groups, with no cases of tongue bleeding. There was no significant difference in tongue swelling at 3 mo between the ADM and PC groups (*p* = 0.845). Results for tongue function revealed lower incidence of tongue-related complications in the ADM group than in the PC group. The most frequent functional complications were tongue numbness, tongue deviation, and eating issues, which were mostly mild in severity. However, there were no significant differences between the groups (*p* > 0.05).

### 3.3 Factors influencing tongue pain after LMGU

ADM use (*p* < 0.0001) and urethral stricture length (*p* = 0.044) had a significant impact on the intensity of early-stage tongue pain. The GLMM results indicate that for every 1-cm increment in LMG length, oral pain increased by a factor of 0.30. The 3-mo results show that the location of the LMG harvest site (*p* = 0.046) and surgical history (*p* = 0.024) had a significant impact on pain at 3 mo. Factors such as age, follow-up time, stricture location and etiology, and LMG length and width had no significant impact on early or 3-mo tongue pain (Table 3).

**Table 3:** Effect of covariates on the intensity of oral pain after LMG harvesting

Covariate	Coefficient	95% CI	*p* value
**Early postoperative pain**			
Age	0.002	−0.033–0.037	0.895
Intervention group (ADM vs PC)	−2.48	−3.571 to −1.380	0.000
Urethral stricture length	0.53	0.013–1.041	0.044
LMG width	0.55	−1.04–2.144	0.497
LMG length	0.30	−0.164–0.767	0.204
LMG harvest site			0.936
Left vs bilateral	0.28	−1.897–2.328	0.729
Right vs bilateral	0.12	−1.537–1.296	0.868
Urethral stricture location	–	–	0.246
Previous interventions			0.955
Minimally invasive vs none	0.15	−1.178–1.469	0.829
Urethroplasty vs none	0.003	−1.538–1.544	0.997
Time to diagnosis	0.01	−0.037–0.059	0.650
Etiology	–	–	0.349
**Pain at 3 mo after surgery**			
Age	0.01	−0.055–0.027	0.647
Intervention group (ADM vs PC)	−0.51	−1.828 to 0.807	0.448
Urethral stricture length	0.02	−0.561–0.523	0.237
LMG width	0.17	−1.791–1.461	0.992
LMG length	0.25	−0.214 to0.711	0.848
LMG harvest site			0.046
Left vs bilateral	−3.02	−5.874 to −0.162	0.038
Right vs bilateral	−1.84	−4.606 to 0.919	0.191
Urethral stricture location	–	–	0.778
Previous interventions			0.024
Minimally invasive vs none	1.81	0.468–3.518	0.008
Urethroplasty vs none	1.61	−0.005 to 3.217	0.051
Time to diagnosis	0.01	−0.044–0.070	0.660
Etiology	–	–	0.800

ADM = acellular dermal matrix; LMG = lingual mucosa graft; PC = primary closure.

### 3.4 Impact of baseline parameters on oral morbidity

Effect sizes in terms of GLMM coefficients indicate that ADM use had a significant impact on early-stage swelling (95% confidence interval for the GLMM coefficient [CI_GLMM_] 0.46–2.32; *p* = 0.003; Supplementary Table 1). There were no cases of tongue bleeding. According to the 3-mo results, the location of the LMG harvest site had a significant impact on tongue numbness (95% CI_GLMM_ −3.21 to −0.37; *p* = 0.014; Supplementary Table 4), impairment of eating and drinking (95% CI_GLMM_ −6.39 to −0.90; *p* = 0.009; Supplementary Table 5), speaking disorders (95% CI_GLMM_ −2.62 to −0.04; *p* = 0.044; Supplementary Table 8), difficulty in tongue protrusion (95% CI_GLMM_ −4.20 to −0.14; *p* = 0.036; Supplementary Table 9), and tongue deviation (95% CI_GLMM_ −3.39 to −0.02; *p* = 0.047; Supplementary Table 10). Bilateral versus unilateral LMG harvesting exacerbated tongue numbness (Supplementary Table 4), impairment of eating and drinking (Supplementary Table 5), speaking disorders (Supplementary Table 8), and tongue deviation (Supplementary Table 10), while left-sided harvesting had a worse effect than right-sided harvesting on these dysfunctions. Numbness was approximately 1.4 times more severe in the left-sided group than in the right-sided group. Left-sided LMG harvesting also had a greater impact on salivary secretion. LMG width affected tongue numbness (95% CI_GLMM_ 1.08–4.06; *p* = 0.001; Supplementary Table 4), taste alteration (95% CI_GLMM_ 0.62–4.04; *p* = 0.008; Supplementary Table 6), speaking disorders (95% CI_GLMM_ 0.16–0.61; *p* = 0.023; Supplementary Table 8), and tongue deviation (95% CI_GLMM_ 1.31–4.48; *p* = 0.001; Supplementary Table 10). For every 1-cm increment in LMG width, numbness increased by a factor of 2.57 (Supplementary Table 4), while speaking disorders(Supplementary Table 8), tongue deviation(Supplementary Table 10), and taste alteration (Supplementary Table 6) increased by factors of 1.49, 3.07, and 2.33, respectively. Age, follow-up time, stricture location, etiology, and LMG length had no significant impact on 3-mo oral morbidity.

## 4 Discussion

LMG has emerged as a reliable and popular graft material for urethral substitution. Simonato et al [13], [14] were the first to describe the use of lingual tissue as an alternative donor site for urethroplasty, and achieved favorable functional and esthetic outcomes. Our team subsequently demonstrated the feasibility of LMG use as a urethral replacement in animal experiments [17]. However, LMG harvesting inevitably causes trauma to the donor site, so the condition of the tongue donor area is an important aspect when evaluating surgical outcomes. Midterm results reported by our team in 2017 for urethroplasty using strip-shaped LMGs showed that patients experienced significant resolution of tongue pain by postoperative day 7 [18], [19]. Our team used lateral LMGs for repair of long-segment urethral strictures and found that nearly half of donor-site complications subsided within 12 mo [20]. Subsequently, numerous studies have explored the impact of donor site morbidity, such as pain, slurred speech, and numbness, on patient-reported quality of life [11], [21], [22]. In all these studies, the donor site was either closed or left open to reduce the wound size and eliminate postoperative bleeding. Despite these oral morbidities, there is still no consensus on management of the donor-site defect.

The Lando ADM used in our study is treated to remove epidermal and cellular components while keeping the basement membrane complex and collagen bundles of the extracellular matrix intact. Results have demonstrated that this ADM is non-immunogenic and enhances neovascularization and fibroblast ingrowth [23], [24]. The DAM comprises a silicone layer and a matrix layer: the upper layer controls moisture loss and is breathable and antibacterial, while the lower layer guides cell growth and gradually degrades. According to Kellner et al [25], the advantage of ADM use in this setting might lie in the ability to harvest broader oral mucosal grafts, especially for long-segment urethral strictures. However, it remains unclear whether ADM use is beneficial for LMG repair. Against this background, this is the first study to compare the effects of PC versus ADM coverage on postoperative pain and morbidity at the graft harvest site for patients undergoing LMGU.

Defining the surgical success of urethroplasty, particularly with oral mucosa grafts, remains challenging. Consensus efforts have revealed widely variable success rates (23–75%) that depend on the criteria used to measure success, which range from isolated urethral patency to comprehensive frameworks [26], [27]. Unlike end-to-end urethral anastomosis, LMGU involves unique donor-site morbidity that impacts oral function. Consequently, LMGU success requires multidimensional assessment beyond urethral patency versus stricture, and should incorporate both lingual-specific functional outcomes and oral complications as essential metrics. Such a comprehensive, multidimensional assessment of LMGU success, featuring outcome reporting across both urological and oral domains, will establish a more patient-centered and pragmatic framework for evaluating postoperative results that can offer valuable guidance for clinical decision-making.

ADM use for coverage and suturing facilitates early patient benefits. Oral pain and swelling are the most significant symptoms reported by patients postoperatively, with >50% of patients experiencing moderate to severe pain, and more than one-third reporting moderate to severe swelling in the early postoperative period. This can be attributed to tension at the wound site and the impact of surgical sutures. We found that ADM use significantly reduced postoperative swelling and pain in comparison to PC, providing early patient benefits. In our study, early postoperative donor site pain and swelling were more severe in the PC group. Several studies have suggested that the graft site should be left open, as this may help in reducing postoperative pain and swelling [10], [12]. Earlier research suggested that PC effectively controls bleeding; however, our study showed no significant difference in tongue bleeding between the ADM and PC groups, which indicates that PC does not offer a significant advantage. According to Jamal et al [28], the advantage of ADM use may lie in allowing harvesting of broader oral mucosal grafts, especially for long-segment urethral strictures.

### 4.1 ADM use has no impact on long-term oral morbidity

LMGs offer a viable alternative to BMGs for urethral reconstruction, with advantages that include less bleeding, easier harvesting, and shorter operative times [5], [22], [29]. While both graft types show comparable urethral success rates and overall pain profiles, they have distinct functional morbidity patterns because of anatomic differences. LMG harvesting causes more early speech/swallowing impairment and taste disturbances (reflecting tongue mobility), whereas BMG harvesting is associated with greater masticatory limitations and long-term risk of trismus/perioral numbness [16], [17], [30].

Notably, donor sites were traditionally sutured closed because of concerns regarding hemostasis and healing. However, growing evidence suggests that leaving the site unclosed can reduce early postoperative pain in masticatory muscles. Our application of ADM to LMG sites aligns with this principle: an ADM provides coverage without tension and can potentially reduce pain and swelling. This technique may further optimize the morbidity profile of LMG harvesting and could help in bridging the early functional gap between LMGs and BMGs. Our findings highlight the importance of graft-specific assessment of the donor site and tailored functional questionnaires. A nuanced understanding of these distinctions is critical for optimizing patient counseling and surgical planning.

The tongue has greater involvement than the buccal muscle in functions such as speech, eating, and drinking. Although morbidity was lower in the ADM group than in the PC group, there were no significant differences between the groups in pain, swelling, or recovery of tongue function at 3 mo after surgery. No cases of tongue bleeding were observed. Our analyses revealed that the location of the harvest site and the LMG width were the most critical factors affecting recovery of tongue function. The location of the harvest site influenced tongue movements, numbness, speaking disorders, tongue deviation, difficulty in tongue protrusion, and impairment of eating and drinking; while LMG width primarily affected sensory perception, including numbness, speaking disorders, tongue deviation, and taste alteration. The tip of the tongue, the front sides, and the middle sides of the tongue are sensitive to sweetness, saltiness, and sourness, respectively. When the lingual mucosa is damaged, injury to taste cells may lead to alterations in taste perception. LMG sampling is primarily conducted on the ventral side of the tongue, but inevitably extends to the mucosal regions of the body of the tongue to obtain a broader graft size, which causes damage to the taste areas. This could be a significant factor affecting tongue function. Lumen et al [6] suggested closing the mucosal edge only when there is minimal tension and leaving the LMG donor site open to reduce restrictions on tongue movement. Chauhan et al [31] recommended leaving a 4–5-mm mucosal margin from the dorsal edge when harvesting free LMGs to prevent damage to the taste buds and preserving at least 1 cm of mucosa from the tip of the tongue to avoid slurred speech. In addition, care should be taken to avoid including underlying tongue muscles and nerves in the graft, as this can lead to contraction, numbness, and greater bleeding.

Although there were no significant differences in 3-mo lingual complications between the two groups, postoperative morphological assessments demonstrated superior outcomes in the ADM group in comparison to the PC group. The ADM group exhibited no significant ventral curvature or deviation of the tongue, with minimal scar contracture at the surgical site. By contrast, the PC group showed marked deviation of the tongue towards the affected side, and prominent scarring (Fig. 1). This discrepancy may be attributed to patients’ functional adaptation to morphological changes in their tongue over time. An ADM can cover the defect area and significantly reduce wound tension, especially for larger harvesting areas.

Our 3-mo postoperative results indicate that bilateral harvesting and the LMG width are key factors affecting tongue function, while ADM use had no significant impact. As a temporary scaffold, ADM physically isolates the wound and reduces traction tension from the teeth and lingual muscles on the exposed wound margins, which thus provides a more stable and comfortable environment for early healing. Therefore, ADM is a suitable choice for complex, long-segment urethral strictures requiring bilateral harvesting or wider LMGs. Conversely, in straightforward cases needing narrow or unilateral LMG harvesting, in which wound tension is minimal and natural healing capacity is sufficient, conventional PC may be adequate.

### 4.2 An understanding of postoperative lingual morbidity after LMG informs evidence-based clinical decision-making

Lingual pain and morbidity, as subjective patient experiences, may be influenced not only by graft harvesting techniques but also by perioperative care practices. An understanding of potential complications following LMGU can guide perioperative care for oral wounds, reduce the incidence of complications, and facilitate early restoration of oral function. For instance, early appropriate analgesia significantly alleviates oral and urethral pain. Iced water or ice cream may reduce initial tongue swelling, and early speech therapy aids in functional recovery [32], [33]. These principles apply not only to urethral reconstruction but also to graft harvesting for other genitourinary reconstructive surgeries. Critically, identical standardized protocols for oral care across both groups in our study allow us to rule out perioperative nursing as a confounding variable in lingual morbidity outcomes.

Although this is the largest clinical study on donor-site pain and morbidity following LMGU to date, it is not without limitations. The main limitations are the retrospective design and the lack of preoperative assessment of the tongue. In addition, long-term follow-up results are subject to recall bias. A fundamental limitation is the potential for historical bias, as the ADM and PC cohorts were treated in different time periods. Despite the use of propensity score matching to balance measurable baseline characteristics, this cannot account for unmeasured temporal confounders that may systematically influence outcomes. These “era effects” include the natural evolution of surgical experience and techniques, advances in perioperative care standards, changes in analgesia protocols, and potential differences in how patient-reported outcome questionnaires were administered over time. In this context, whereby overall surgical and perioperative management tends to improve over time, it is plausible that the ADM group may have had better outcomes irrespective of the intervention studied. The questionnaire response rate was 55.8% (250/448), which raises the possibility that respondents may not be fully representative of the entire cohort. For instance, patients with poorer outcomes or lower satisfaction might have been less inclined to respond, which could potentially lead to overestimation of positive patient-reported results. However, there is currently no validated questionnaire specifically addressing oral morbidity after LMGU. Therefore, an effective questionnaire for assessing oral morbidity following LMGU should be developed in future studies.

## 5 Conclusions

ADM use for coverage during suturing after LMG harvesting provides early patient benefits by significantly reducing swelling and pain. Long-term outcomes are comparable to those for PC.

  ***Author contributions:*** Qiang Fu and Lujie Song had full access to all the data in the study and take responsibility for the integrity of the data and the accuracy of the data analysis.

  *Study concept and design*: Song, Fu, Hou.

*Acquisition of data*: Hou, Yu, Liu, Liang, Ye, Jin, Sa, Si.

*Analysis and interpretation of data*: Hou, Zhu, Dai.

*Drafting of the manuscript*: Hou, Song, Yu, Fu.

*Critical revision of the manuscript for important intellectual content*: Song, Fu.

*Statistical analysis*: Hou, Zhu, Song.

*Obtaining funding*: Fu, Song.

*Administrative, technical, or material support*: None.

*Supervision*: Fu, Song.

*Other*: None.

  ***Financial disclosures:*** Qiang Fu certifies that all conflicts of interest, including specific financial interests and relationships and affiliations relevant to the subject matter or materials discussed in the manuscript (eg, employment/affiliation, grants or funding, consultancies, honoraria, stock ownership or options, expert testimony, royalties, or patents filed, received, or pending), are the following: None.

  ***Funding/Support and role of the sponsor:*** This study was supported by grants from the Discipline Leader of Shanghai Municipal Health program (grant 2022XD015), the Summit Plateau Program, the Research Physician Program, Shanghai Jiao Tong University School of Medicine (grant 20240817), and the Shanghai Science and Technology Innovation Action Plan (grant 22Y11905000). The funding bodies played a role in the design and conduct of the study; collection, management, analysis, and interpretation of the data; and preparation, review, and approval of the manuscript.

  ***Ethics statement:*** This study was approved by the ethics committee of Shanghai Sixth People’s Hospital (reference 2022-KY-065).
