# Supplementary material for: Impact of Coverage with an Acellular Dermal Matrix and Suturing Versus Primary Closure on Tongue Pain and Oral Morbidity After Lingual Mucosa Harvesting for Urethroplasty: A Retrospective Cohort Study
Source: Eur Urol Open Sci. 2025 Dec 10;83:109–18. doi: 10.1016/j.euros.2025.11.010 (PMC12755985; doi:10.1016/j.euros.2025.11.010)
Supplement: Supplementary Data 1 [file mmc1.docx]

**The post-lingual mucosa urethroplasty tongue health questionnaire**

**Early-stage questionnaire**

1. Short-Form McGill Pain Questionnaire Present Pain Intensity (SF-MPQ-PPI)：How would you rate your tongue pain intensity during the first 7 days following lingual mucosal graft harvesting surgery?

□ 0 No pain

□ 1 Mild

□ 2 Discomforting

□ 3 Distressing

□ 4 Horrible

□ 5 Excruciating

1. How would you rate your tongue bleeding intensity during the first 7 days following lingual mucosal graft harvesting surgery?

□ 0 None at all

□ 1 Slight

□ 2 Moderate

□ 3 Severe

□ 4 Very Severe

1. How would you rate your tongue swelling intensity during the first 7 days following lingual mucosal graft harvesting surgery?

□ 0 None at all

□ 1 Slight

□ 2 Moderate

□ 3 Severe

□ 4 Very Severe

**Long-term questionnaire**

1. Short-Form McGill Pain Questionnaire Present Pain Intensity (SF-MPQ-PPI)：How would you describe the intensity of your oral pain at the 3-month follow-up after lingual mucosa harvest

□ 0 No pain

□ 1 Mild

□ 2 Discomforting

□ 3 Distressing

□ 4 Horrible

□ 5 Excruciating

1. How would you describe the intensity of your tongue bleeding at the 3-month follow-up after lingual mucosa harvest

□ 0 None at all

□ 1 Slight

□ 2 Moderate

□ 3 Severe

□ 4 Very Severe

1. How would you describe the intensity of your tongue swelling at the 3-month follow-up after lingual mucosa harvest

□ 0 None at all

□ 1 Slight

□ 2 Moderate

□ 3 Severe

□ 4 Very Severe

1. How would you describe the intensity of your tongue numbness at the 3-month follow-up after lingual mucosa harvest

□ 0 None at all

□ 1 Slight

□ 2 Moderate

□ 3 Severe

□ 4 Very Severe

1. How would you describe the intensity of your impairment of eating and drinking at the 3-month follow-up after lingual mucosa harvest

□ 0 None at all

□ 1 Slight

□ 2 Moderate

□ 3 Severe

□ 4 Very Severe

1. How would you describe the intensity of your alteration of taste perception at the 3-month follow-up after lingual mucosa harvest

□ 0 None at all

□ 1 Slight

□ 2 Moderate

□ 3 Severe

□ 4 Very Severe

1. How would you describe the intensity of your alteration of salivation at the 3-month follow-up after lingual mucosa harvest

□ 0 None at all

□ 1 Slight

□ 2 Moderate

□ 3 Severe

□ 4 Very Severe

1. How would you describe the intensity of your speaking disorders at the 3-month follow-up after lingual mucosa harvest

□ 0 None at all

□ 1 Slight

□ 2 Moderate

□ 3 Severe

□ 4 Very Severe

1. How would you describe the intensity of your speaking disorders at the 3-month follow-up after lingual mucosa harvest

□ 0 None at all

□ 1 Slight

□ 2 Moderate

□ 3 Severe

□ 4 Very Severe

1. How would you describe the intensity of your difficulty in tongue protrusion at the 3-month follow-up after lingual mucosa harvest

□ 0 None at all

□ 1 Slight

□ 2 Moderate

□ 3 Severe

□ 4 Very Severe

1. How would you describe the intensity of the degree of tongue deviation or retraction at the 3-month follow-up after lingual mucosa harvest

□ 0 None at all

□ 1 Slight

□ 2 Moderate

□ 3 Severe

□ 4 Very Severe
