# Supplementary material for: Impact of Coverage with an Acellular Dermal Matrix and Suturing Versus Primary Closure on Tongue Pain and Oral Morbidity After Lingual Mucosa Harvesting for Urethroplasty: A Retrospective Cohort Study
Source: Eur Urol Open Sci. 2025 Dec 10;83:109–18. doi: 10.1016/j.euros.2025.11.010 (PMC12755985; doi:10.1016/j.euros.2025.11.010)
Supplement: Supplementary Data 2 [file mmc2.docx]

**Supplement Tables-Covariates and their effect on intensity of oral morbidity after LMGU**

| **Supplementary Table 1. Early-stage Tongue Swelling** | | | | | | |
| --- | --- | --- | --- | --- | --- | --- |
|  | |  | **Coefficient** | ***P*** | **95% CI** | |
|  | |  |  |  | **Lower-bound** | **Upper-bound** |
| **Covariate** | **Age** |  | 0.002 | 0.903 | 0.970 | 1.036 |
|  | **Time to diagnosis** |  | 0.011 | 0.166 | -0.037 | 0.059 |
|  | **Length of the urethral stricture** | | -0.017 | 0.930 | -0.406 | 0.371 |
|  | **Length of Lingual mucosa graft** | | -0.152 | 0.407 | -0.512 | 0.207 |
|  | **Width of Lingual mucosa graft** | | 1.612 | 0.008 | 0.418 | 2.806 |
|  | **Groups** | **ADM Group** | 0^a^ |  |  |  |
|  |  | **PC Group** | 1.390 | 0.003 | 1.588 | 10.153 |
|  | **Lingual mucosa graft harvest site** | **Left** | -0.085 | 0.900 | 0.900 | 0.900 |
|  |  | **Right** | -0.770 | 0.217 | 0.217 | 0.217 |
|  |  | **Bilateral** | 0^a^ |  |  |  |
|  | **Location of the urethral stricture** | **S1** | 20.592 | 0.999 | -67012.501 | 67053.686 |
|  |  | **S2a** | 22.496 | 0.999 | -67010.598 | 67055.590 |
|  |  | **S2b** | 20.995 | 0.999 | -67012.098 | 67054.089 |
|  |  | **S2c** | 21.282 | 0.999 | -67011.811 | 67054.376 |
|  |  | **S2d** | 19.787 | 0.999 | -67013.307 | 67052.881 |
|  |  | **S2d+S1** | -0.168 | 1.000 | -107942.122 | 107941.787 |
|  |  | **S2d+S2b** | 44.164 | 0.999 | -105701.862 | 105790.191 |
|  |  | **S3** | 0^a^ |  |  |  |
|  | **Previous interventions** | **Non** | -0.226 | 0.810 | -1.342 | 0.890 |
|  |  | **Minimally invasive** | 0.093 | 0.691 | 0.411 | 3.825 |
|  |  | **Urethralplasty** | 0^a^ |  |  |  |
|  | **Etiology** | **Iatrogenic** | -24.208 | 1.000 | -108115.900 | 108067.484 |
|  |  | **Congenital** | -22.503 | 1.000 | -108114.195 | 108069.188 |
|  |  | **Traumatic** | -22.599 | 1.000 | -108114.291 | 108069.092 |
|  |  | **Lichen sclerosus** | -23.667 | 1.000 | -108115.358 | 108068.025 |
|  |  | **Idiopathic/unknown** | -23.757 | 1.000 | -108115.449 | 108067.935 |
|  |  | **Failed hypospadias repair** | -24.260 | 1.000 | -108115.952 | 108067.432 |
|  |  | **Hypospadias** | -43.756 | 1.000 | -189621.161 | 189533.649 |
|  |  | **Inflammatory** | 0^a^ |  |  |  |

| **Supplementary Table 2. Early-stage Tongue Bleeding** | | | | | | |
| --- | --- | --- | --- | --- | --- | --- |
|  | |  | **Coefficient** | ***P*** | **95% CI** | |
|  | |  |  |  | **Lower-bound** | **Upper-bound** |
| **Covariate** | **Age** | | -0.017 | 0.166 | -0.041 | 0.007 |
|  | **Time to diagnosis** | | -0.008 | 0.651 | -0.042 | 0.026 |
|  | **Length of the urethral stricture** | | -0.017 | -0.406 | 0.371 | 0.930 |
|  | **Length of Lingual mucosa graft** | | -0.152 | -0.512 | 0.207 | 0.407 |
|  | **Width of Lingual mucosa graft** | | 1.612 | 0.418 | 2.806 | 0.008 |
|  | **Groups** | **ADM Group** | -0.155 | 0.681 | -0.895 | 0.585 |
|  |  | **PC Group** | 0^a^ |  |  |  |
|  | **Lingual mucosa graft harvest site** | **Left** | 0.549 | 0.339 | -0.577 | 1.674 |
|  |  | **Right** | 0.286 | 0.584 | -0.739 | 1.311 |
|  |  | **Bilateral** | 0^a^ |  |  |  |
|  | **Location of the urethral stricture** | **S1** | 0.292 | 0.823 | -2.260 | 2.844 |
|  |  | **S2a** | 1.222 | 0.406 | -1.661 | 4.104 |
|  |  | **S2b** | 0.500 | 0.698 | -2.023 | 3.023 |
|  |  | **S2c** | 0.824 | 0.606 | -2.306 | 3.954 |
|  |  | **S2d** | -0.800 | 0.581 | -3.637 | 2.038 |
|  |  | **S2d+S1** | -0.240 | 0.895 | -3.820 | 3.340 |
|  |  | **S2d+S2b** | 24.830 | 0.823 | -85337.932 | 85387.593 |
|  |  | **S3** | 0^a^ |  |  |  |
|  | **Previous interventions** | **Non** | 0.747 | 0.094 | -0.128 | 1.621 |
|  |  | **Minimally invasive** | 0.599 | 0.275 | -0.475 | 1.673 |
|  |  | **Urethralplasty** | 0^a^ |  |  |  |
|  | **Etiology** | **Iatrogenic** | 22.064 | 1.000 | -109953.933 | 109998.061 |
|  |  | **Congenital** | 22.499 | 1.000 | -109953.498 | 109998.496 |
|  |  | **Traumatic** | 22.055 | 1.000 | -109953.942 | 109998.052 |
|  |  | **Lichen sclerosus** | 21.197 | 1.000 | -109954.800 | 109997.194 |
|  |  | **Idiopathic/unknown** | 22.183 | 1.000 | -109953.814 | 109998.180 |
|  |  | **Failed hypospadias repair** | 20.949 | 1.000 | -109955.048 | 109996.946 |
|  |  | **Hypospadias** | 45.105 | 1.000 | -190612.943 | 190703.153 |
|  |  | **Inflammatory** | 0^a^ |  |  |  |

| **Supplementary Table 3. Long-stage Tongue Swelling** | | | | | | |
| --- | --- | --- | --- | --- | --- | --- |
|  | |  | **Coefficient** | ***P*** | **95% CI** | |
|  | |  |  |  | **Lower-bound** | **Upper-bound** |
| **Covariate** | **Age** | | -0.011 | 0.647 | -0.056 | 0.035 |
|  | **Time to diagnosis** | | -0.019 | 0.512 | -0.074 | 0.037 |
|  | **Length of the urethral stricture** | | -0.474 | 0.237 | -1.260 | 0.312 |
|  | **Length of Lingual mucosa graft** | | 0.068 | 0.848 | -0.628 | 0.764 |
|  | **Width of Lingual mucosa graft** | | 0.009 | 0.992 | -1.888 | 1.907 |
|  | **Groups** | **ADM Group** | -0.087 | 0.906 | -1.531 | 1.358 |
|  |  | **PC Group** | 0^a^ |  |  |  |
|  | **Lingual mucosa graft harvest site** | **Left** | -22.284 | 0.998 | -21634.027 | 21589.459 |
|  |  | **Right** | -21.742 | 0.998 | -21633.485 | 21590.001 |
|  |  | **Bilateral** | 0^a^ |  |  |  |
|  | **Location of the urethral stricture** | **S1** | 2.652 | 0.150 | -0.960 | 6.264 |
|  |  | **S2a** | 22.641 | 0.999 | -52836.960 | 52882.243 |
|  |  | **S2b** | 2.985 | 0.113 | -0.709 | 6.679 |
|  |  | **S2c** | 23.689 | 0.999 | -65111.401 | 65158.780 |
|  |  | **S2d** | 1.271 | 0.526 | -2.659 | 5.201 |
|  |  | **S2d+S1** | .954 | 0.656 | -3.240 | 5.148 |
|  |  | **S2d+S2b** | 23.982 | 1.000 | -75938.643 | 75986.606 |
|  |  | **S3** | 0^a^ |  |  |  |
|  | **Previous interventions** | **Non** | 1.947 | 0.007 | 0.521 | 3.373 |
|  |  | **Minimally invasive** | 1.467 | 0.100 | -0.281 | 3.216 |
|  |  | **Urethralplasty** | 0^a^ |  | . | . |
|  | **Etiology** | **Iatrogenic** | -20.882 | 1.000 | -94621.009 | 94579.245 |
|  |  | **Congenital** | -19.398 | 1.000 | -94619.525 | 94580.728 |
|  |  | **Traumatic** | -20.580 | 1.000 | -94620.706 | 94579.547 |
|  |  | **Lichen sclerosus** | -19.711 | 1.000 | -94619.838 | 94580.416 |
|  |  | **Idiopathic/unknown** | -20.070 | 1.000 | -94620.197 | 94580.057 |
|  |  | **Failed hypospadias repair** | -22.177 | 1.000 | -94622.304 | 94577.950 |
|  |  | **Hypospadias** | 0.497 | 1.000 | -182221.772 | 182222.766 |
|  |  | **Inflammatory** | 0^a^ |  |  |  |

| **Supplementary Table 4. Long-stage Tongue Numbness** | | | | | | |
| --- | --- | --- | --- | --- | --- | --- |
|  | |  | **Coefficient** | ***P*** | **95% CI** | |
|  | |  |  |  | **Lower-bound** | **Upper-bound** |
| **Covariate** | **Age** | | -0.005 | 0.740 | -0.031 | 0.022 |
|  | **Time to diagnosis** | | 0.008 | 0.693 | -0.030 | 0.046 |
|  | **Length of the urethral stricture** | | -0.109 | 0.617 | -0.536 | 0.318 |
|  | **Length of Lingual mucosa graft** | | 0.109 | 0.617 | 0.727 | 1.709 |
|  | **Width of Lingual mucosa graft** | | 2.572 | 0.001 | 2.956 | 57.987 |
|  | **Groups** | **ADM Group** | 0.315 | 0.906 | -1.531 | 1.358 |
|  |  | **PC Group** | 0^a^ |  |  |  |
|  | **Lingual mucosa graft harvest site** | **Left** | -1.789 | 0.014 | -3.208 | -0.369 |
|  |  | **Right** | -0.641 | 0.339 | -1.954 | 0.673 |
|  |  | **Bilateral** | 0^a^ |  |  |  |
|  | **Location of the urethral stricture** | **S1** | 1.000 | 0.150 | -.960 | 6.264 |
|  |  | **S2a** | 0.520 | 0.999 | -52836.960 | 52882.243 |
|  |  | **S2b** | 1.359 | 0.113 | -0.709 | 6.679 |
|  |  | **S2c** | 22.701 | 0.999 | -65111.401 | 65158.780 |
|  |  | **S2d** | 0.392 | 0.526 | -2.659 | 5.201 |
|  |  | **S2d+S1** | 1.517 | 0.656 | -3.240 | 5.148 |
|  |  | **S2d+S2b** | 1.411 | 1.000 | -75938.643 | 75986.606 |
|  |  | **S3** | 0^a^ |  |  |  |
|  | **Previous interventions** | **Non** | 0.304 | 0.007 | 0.521 | 3.373 |
|  |  | **Minimally invasive** | -0.744 | 0.100 | -0.281 | 3.216 |
|  |  | **Urethralplasty** | 0^a^ |  | . | . |
|  | **Etiology** | **Iatrogenic** | 0.875 | 1.000 | -94621.009 | 94579.245 |
|  |  | **Congenital** | 0.899 | 1.000 | -94619.525 | 94580.728 |
|  |  | **Traumatic** | 1.002 | 1.000 | -94620.706 | 94579.547 |
|  |  | **Lichen sclerosus** | 1.843 | 1.000 | -94619.838 | 94580.416 |
|  |  | **Idiopathic/unknown** | 1.984 | 1.000 | -94620.197 | 94580.057 |
|  |  | **Failed hypospadias repair** | 0.660 | 1.000 | -94622.304 | 94577.950 |
|  |  | **Hypospadias** | 21.991 | 1.000 | -182221.772 | 182222.766 |
|  |  | **Inflammatory** | 0^a^ |  |  |  |

| **Supplementary Table 5. Impairment of eating and drinking** | | | | | | |
| --- | --- | --- | --- | --- | --- | --- |
|  | |  | **Coefficient** | ***P*** | **95% CI** | |
|  | |  |  |  | **Lower-bound** | **Upper-bound** |
| **Covariate** | **Age** | | -0.010 | 0.573 | -0.045 | 0.025 |
|  | **Time to diagnosis** | | 0.013 | 0.616 | -0.037 | 0.062 |
|  | **Length of the urethral stricture** | | -0.447 | 0.242 | -1.196 | 0.302 |
|  | **Length of Lingual mucosa graft** | | 0.176 | 0.617 | -0.514 | 0.866 |
|  | **Width of Lingual mucosa graft** | | -0.355 | 0.665 | -1.960 | 1.250 |
|  | **Groups** | **ADM Group** | 0.764 | 0.906 | -1.531 | 0.180 |
|  |  | **PC Group** | 0^a^ |  |  | . |
|  | **Lingual mucosa graft harvest site** | **Left** | -3.644 | 0.009 | -6.390 | -0.898 |
|  |  | **Right** | -2.107 | 0.115 | -4.726 | 0.513 |
|  |  | **Bilateral** | 0^a^ |  |  |  |
|  | **Location of the urethral stricture** | **S1** | 2.492 | 0.103 | -0.499 | 5.483 |
|  |  | **S2a** | 0.707 | 0.679 | -2.644 | 4.059 |
|  |  | **S2b** | 1.388 | 0.308 | -1.279 | 4.055 |
|  |  | **S2c** | 0.877 | 0.636 | -2.752 | 4.506 |
|  |  | **S2d** | -0.221 | 0.884 | -3.192 | 2.750 |
|  |  | **S2d+S1** | 22.218 | 1.000 | -85527.643 | 85572.079 |
|  |  | **S2d+S2b** | 22.516 | 1.000 | -85180.359 | 85225.390 |
|  |  | **S3** | 0^a^ |  |  |  |
|  | **Previous interventions** | **Non** | 1.619 | 0.015 | 0.317 | 2.920 |
|  |  | **Minimally invasive** | 0.919 | 0.200 | -0.486 | 2.323 |
|  |  | **Urethralplasty** | 0^a^ |  | . | . |
|  | **Etiology** | **Iatrogenic** | -21.432 | 1.000 | -100419.283 | 100376.419 |
|  |  | **Congenital** | -20.940 | 1.000 | -100418.791 | 100376.911 |
|  |  | **Traumatic** | -21.299 | 1.000 | -100419.150 | 100376.552 |
|  |  | **Lichen sclerosus** | -19.615 | 1.000 | -100417.466 | 100378.236 |
|  |  | **Idiopathic/unknown** | -19.988 | 1.000 | -100417.839 | 100377.863 |
|  |  | **Failed hypospadias repair** | -20.949 | 1.000 | -100418.801 | 100376.902 |
|  |  | **Hypospadias** | 0.061 | 1.000 | -185298.346 | 185298.469 |
|  |  | **Inflammatory** | 0^a^ |  |  |  |

| **Supplementary Table 6. Alteration of taste perception** | | | | | | |
| --- | --- | --- | --- | --- | --- | --- |
|  | |  | **Coefficient** | ***P*** | **95% CI** | |
|  | |  |  |  | **Lower-bound** | **Upper-bound** |
| **Covariate** | **Age** | | -0.002 | 0.907 | -0.038 | 0.034 |
|  | **Time to diagnosis** | | -0.027 | 0.258 | -0.074 | 0.020 |
|  | **Length of the urethral stricture** | | 0.070 | 0.769 | -0.395 | 0.534 |
|  | **Length of Lingual mucosa graft** | | 0.347 | 0.103 | 0.932 | 2.147 |
|  | **Width of Lingual mucosa graft** | | 2.330 | 0.008 | 1.859 | 56.794 |
|  | **Groups** | **ADM Group** | 0.610 | 0.906 | -1.531 | 0.180 |
|  |  | **PC Group** | 0^a^ |  |  | . |
|  | **Lingual mucosa graft harvest site** | **Left** | -0.828 | 0.329 | -2.488 | 0.833 |
|  |  | **Right** | 0.158 | 0.845 | -1.429 | 1.746 |
|  |  | **Bilateral** | 0^a^ |  |  |  |
|  | **Location of the urethral stricture** | **S1** | -0.002 | 0.999 | -2.800 | 2.796 |
|  |  | **S2a** | -0.280 | 0.871 | -3.653 | 3.093 |
|  |  | **S2b** | -0.033 | 0.981 | -2.690 | 2.624 |
|  |  | **S2c** | 20.681 | 1.000 | -65250.683 | 65292.045 |
|  |  | **S2d** | 0.656 | 0.697 | -2.648 | 3.961 |
|  |  | **S2d+S1** | -1.522 | 0.455 | -5.509 | 2.466 |
|  |  | **S2d+S2b** | 22.727 | 1.000 | -87003.818 | 87049.272 |
|  |  | **S3** | 0^a^ |  |  |  |
|  | **Previous interventions** | **Non** | 1.651 | 0.008 | 0.430 | 2.872 |
|  |  | **Minimally invasive** | 1.256 | 0.088 | -0.188 | 2.700 |
|  |  | **Urethralplasty** | 0^a^ |  | . | . |
|  | **Etiology** | **Iatrogenic** | 0.010 | 0.996 | -3.407 | 3.427 |
|  |  | **Congenital** | 0.869 | 0.620 | -2.565 | 4.303 |
|  |  | **Traumatic** | -.073 | 0.969 | -3.716 | 3.569 |
|  |  | **Lichen sclerosus** | .462 | 0.797 | -3.058 | 3.983 |
|  |  | **Idiopathic/unknown** | 1.381 | 0.418 | -1.962 | 4.724 |
|  |  | **Failed hypospadias repair** | 1.007 | 0.560 | -2.382 | 4.395 |
|  |  | **Hypospadias** | 21.415 | 1.000 | -155721.230 | 155764.059 |
|  |  | **Inflammatory** | 0^a^ |  |  |  |

| **Supplementary Table 7. Alteration of salivation** | | | | | | |
| --- | --- | --- | --- | --- | --- | --- |
|  | |  | **Coefficient** | ***P*** | **95% CI** | |
|  | |  |  |  | **Lower-bound** | **Upper-bound** |
| **Covariate** | **Age** | | 0.003 | 0.884 | -0.038 | 0.044 |
|  | **Time to diagnosis** | | -0.015 | 0.638 | -0.075 | 0.046 |
|  | **Length of the urethral stricture** | | -0.130 | 0.646 | -0.682 | 0.423 |
|  | **Length of Lingual mucosa graft** | | -0.371 | 0.129 | -0.851 | 0.109 |
|  | **Width of Lingual mucosa graft** | | -1.708 | 0.084 | -3.645 | 0.229 |
|  | **Groups** | **ADM Group** | 0.203 | 0.783 | -1.238 | 1.643 |
|  |  | **PC Group** | 0^a^ |  |  | . |
|  | **Lingual mucosa graft harvest site** | **Left** | -2.919 | 0.030 | -5.557 | -0.281 |
|  |  | **Right** | -0.930 | 0.845 | -1.429 | 1.746 |
|  |  | **Bilateral** | 0^a^ |  |  |  |
|  | **Location of the urethral stricture** | **S1** | 2.127 | 0.145 | -0.731 | 4.985 |
|  |  | **S2a** | 1.563 | 0.378 | -1.908 | 5.033 |
|  |  | **S2b** | 2.176 | 0.111 | -0.501 | 4.853 |
|  |  | **S2c** | 0.731 | 0.710 | -3.117 | 4.579 |
|  |  | **S2d** | 0.857 | 0.577 | -2.152 | 3.866 |
|  |  | **S2d+S1** | 22.474 | 1.000 | -86353.008 | 86397.955 |
|  |  | **S2d+S2b** | 22.580 | 1.000 | -89766.337 | 89811.497 |
|  |  | **S3** | 0^a^ |  |  |  |
|  | **Previous interventions** | **Non** | 1.248 | 0.099 | -0.236 | 2.733 |
|  |  | **Minimally invasive** | 1.771 | 0.068 | -0.130 | 3.671 |
|  |  | **Urethralplasty** | 0^a^ |  | . | . |
|  | **Etiology** | **Iatrogenic** | -22.111 | 1.000 | -106638.246 | 106594.024 |
|  |  | **Congenital** | -20.228 | 1.000 | -106636.363 | 106595.907 |
|  |  | **Traumatic** | -20.903 | 1.000 | -106637.038 | 106595.232 |
|  |  | **Lichen sclerosus** | -21.016 | 1.000 | -106637.151 | 106595.119 |
|  |  | **Idiopathic/unknown** | -20.753 | 1.000 | -106636.888 | 106595.382 |
|  |  | **Failed hypospadias repair** | -20.769 | 1.000 | -106636.904 | 106595.366 |
|  |  | **Hypospadias** | -1.256 | 1.000 | -188741.214 | 188738.701 |
|  |  | **Inflammatory** | 0^a^ |  |  |  |

| **Supplementary Table 8. Speaking disorders** | | | | | | |
| --- | --- | --- | --- | --- | --- | --- |
|  | |  | **Coefficient** | ***P*** | **95% CI** | |
|  | |  |  |  | **Lower-bound** | **Upper-bound** |
| **Covariate** | **Age** | | -0.005 | 0.707 | -0.033 | 0.023 |
|  | **Time to diagnosis** | | -0.006 | 0.751 | -0.042 | 0.030 |
|  | **Length of the urethral stricture** | | -0.041 | 0.850 | -0.467 | 0.385 |
|  | **Length of Lingual mucosa graft** | | 1.493 | 0.023 | 1.224 | 16.165 |
|  | **Width of Lingual mucosa graft** | | 0.228 | 0.246 | 0.855 | 1.845 |
|  | **Groups** | **ADM Group** | 0.301 | 0.783 | -1.238 | 1.643 |
|  |  | **PC Group** | 0^a^ |  |  | . |
|  | **Lingual mucosa graft harvest site** | **Left** | -1.331 | 0.044 | -2.623 | -0.039 |
|  |  | **Right** | -0.099 | 0.872 | -1.314 | 1.115 |
|  |  | **Bilateral** | 0^a^ |  |  |  |
|  | **Location of the urethral stricture** | **S1** | 0.648 | 0.571 | -1.594 | 2.890 |
|  |  | **S2a** | 1.169 | 0.445 | -1.832 | 4.169 |
|  |  | **S2b** | 0.442 | 0.688 | -1.713 | 2.598 |
|  |  | **S2c** | 1.180 | 0.480 | -2.095 | 4.455 |
|  |  | **S2d** | 0.883 | 0.507 | -1.727 | 3.493 |
|  |  | **S2d+S1** | 22.899 | 1.000 | -85988.543 | 86034.341 |
|  |  | **S2d+S2b** | -0.035 | 0.984 | -3.423 | 3.352 |
|  |  | **S3** | 0^a^ |  |  |  |
|  | **Previous interventions** | **Non** | 0.695 | 0.160 | -0.275 | 1.665 |
|  |  | **Minimally invasive** | -0.378 | 0.517 | -1.523 | 0.767 |
|  |  | **Urethralplasty** | 0^a^ |  | . | . |
|  | **Etiology** | **Iatrogenic** | 0.385 | 0.810 | -2.757 | 3.526 |
|  |  | **Congenital** | 1.155 | 0.481 | -2.057 | 4.366 |
|  |  | **Traumatic** | -0.041 | 0.980 | -3.315 | 3.233 |
|  |  | **Lichen sclerosus** | 1.000 | 0.546 | -2.247 | 4.246 |
|  |  | **Idiopathic/unknown** | 0.067 | 0.966 | -3.009 | 3.143 |
|  |  | **Failed hypospadias repair** | -0.368 | 0.816 | -3.465 | 2.729 |
|  |  | **Hypospadias** | 21.652 | 1.000 | -155720.993 | 155764.297 |
|  |  | **Inflammatory** | 0^a^ |  |  |  |

| **Supplementary Table 9. Difficulty in tongue protrusion** | | | | | | |
| --- | --- | --- | --- | --- | --- | --- |
|  | |  | **Coefficient** | ***P*** | **95% CI** | |
|  | |  |  |  | **Lower-bound** | **Upper-bound** |
| **Covariate** | **Age** | | 0.003 | 0.891 | -0.033 | 0.038 |
|  | **Time to diagnosis** | | 0.001 | 0.958 | -0.046 | 0.048 |
|  | **Length of the urethral stricture** | | -0.303 | 0.258 | -0.828 | 0.222 |
|  | **Length of Lingual mucosa graft** | | -0.141 | 0.555 | -0.609 | 0.327 |
|  | **Width of Lingual mucosa graft** | | -1.135 | 0.185 | -2.814 | 0.543 |
|  | **Groups** | **ADM Group** | 0.255 | 0.649 | -0.843 | 1.353 |
|  |  | **PC Group** | 0^a^ |  | . | . |
|  | **Lingual mucosa graft harvest site** | **Left** | -2.170 | 0.036 | -4.199 | -0.141 |
|  |  | **Right** | -1.296 | 0.186 | -3.219 | 0.626 |
|  |  | **Bilateral** | 0^a^ |  |  |  |
|  | **Location of the urethral stricture** | **S1** | 0.955 | 0.504 | -1.846 | 3.756 |
|  |  | **S2a** | 0.649 | 0.704 | -2.693 | 3.991 |
|  |  | **S2b** | 0.167 | 0.899 | -2.413 | 2.747 |
|  |  | **S2c** | 0.505 | 0.783 | -3.080 | 4.090 |
|  |  | **S2d** | -0.422 | 0.776 | -3.324 | 2.481 |
|  |  | **S2d+S1** | 20.822 | 1.000 | -83555.036 | 83596.680 |
|  |  | **S2d+S2b** | 22.171 | 1.000 | -87376.505 | 87420.847 |
|  |  | **S3** | 0^a^ |  |  |  |
|  | **Previous interventions** | **Non** | 1.283 | 0.037 | 0.076 | 2.490 |
|  |  | **Minimally invasive** | 1.907 | 0.027 | 0.213 | 3.601 |
|  |  | **Urethralplasty** | 0^a^ | 0.851 | -3.292 | 3.993 |
|  | **Etiology** | **Iatrogenic** | 0.868 | 0.531 | -2.443 | 4.741 |
|  |  | **Congenital** | 1.761 | 0.276 | -1.484 | 5.202 |
|  |  | **Traumatic** | 0.350 | 0.210 | -1.347 | 6.140 |
|  |  | **Lichen sclerosus** | 1.149 | 1.000 | -155719.908 | 155765.381 |
|  |  | **Idiopathic/unknown** | 1.859 | 0.966 | -3.009 | 3.143 |
|  |  | **Failed hypospadias repair** | 2.397 | 0.816 | -3.465 | 2.729 |
|  |  | **Hypospadias** | 22.737 | 1.000 | -155720.993 | 155764.297 |
|  |  | **Inflammatory** | 0^a^ |  |  |  |

| **Supplementary Table 10. Tongue is skewed or retracted** | | | | | | |
| --- | --- | --- | --- | --- | --- | --- |
|  | |  | **Coefficient** | ***P*** | **95% CI** | |
|  | |  |  |  | **Lower-bound** | **Upper-bound** |
| **Covariate** | **Age** | | 0.016 | 0.330 | -0.016 | 0.049 |
|  | **Time to diagnosis** | | 0.018 | 0.448 | -0.028 | 0.064 |
|  | **Length of the urethral stricture** | | -0.436 | 0.075 | -0.915 | 0.043 |
|  | **Length of Lingual mucosa graft** | | 0.058 | 0.789 | -0.370 | 0.487 |
|  | **Width of Lingual mucosa graft** | | 3.073 | 0.001 | 3.693 | 126.427 |
|  | **Groups** | **ADM Group** | 0.471 | 0.328 | -0.472 | 1.414 |
|  |  | **PC Group** | 0^a^ |  | . | . |
|  | **Lingual mucosa graft harvest site** | **Left** | -1.703 | 0.047 | -3.384 | -0.022 |
|  |  | **Right** | -1.315 | 0.098 | -2.872 | 0.242 |
|  |  | **Bilateral** | 0^a^ |  |  |  |
|  | **Location of the urethral stricture** | **S1** | 1.298 | 0.326 | -1.295 | 3.892 |
|  |  | **S2a** | 22.103 | 0.999 | -52363.254 | 52407.459 |
|  |  | **S2b** | 0.765 | 0.545 | -1.714 | 3.244 |
|  |  | **S2c** | 22.158 | 0.999 | -66876.562 | 66920.879 |
|  |  | **S2d** | 0.387 | 0.787 | -2.426 | 3.201 |
|  |  | **S2d+S1** | 23.022 | 1.000 | -82866.164 | 82912.207 |
|  |  | **S2d+S2b** | 23.511 | 1.000 | -87376.505 | 87420.847 |
|  |  | **S3** | 0^a^ |  |  |  |
|  | **Previous interventions** | **Non** | 0.138 | 0.807 | -0.972 | 1.249 |
|  |  | **Minimally invasive** | 0.080 | 0.914 | -1.368 | 1.529 |
|  |  | **Urethralplasty** | 0^a^ | 0.851 | -3.292 | 3.993 |
|  | **Etiology** | **Iatrogenic** | 1.442 | .405 | -1.952 | 4.836 |
|  |  | **Congenital** | 1.937 | .274 | -1.533 | 5.406 |
|  |  | **Traumatic** | 1.726 | .361 | -1.974 | 5.427 |
|  |  | **Lichen sclerosus** | 1.689 | .345 | -1.819 | 5.196 |
|  |  | **Idiopathic/unknown** | 1.816 | .280 | -1.476 | 5.107 |
|  |  | **Failed hypospadias repair** | 0.377 | 0.819 | -2.857 | 3.610 |
|  |  | **Hypospadias** | 22.874 | 1.000 | -155719.771 | 155765.518 |
|  |  | **Inflammatory** | 0^a^ |  |  |  |
